# Influence of climate and river level on the incidence of malaria in Cacao, French Guiana

**DOI:** 10.1186/1475-2875-10-26

**Published:** 2011-02-04

**Authors:** Célia Basurko, Matthieu Hanf, René Han-Sze, Stéphanie Rogier, Philippe Héritier, Claire Grenier, Michel Joubert, Mathieu Nacher, Bernard Carme

**Affiliations:** 1 Centre d'Investigation Clinique Epidémiologie Clinique Antilles Guyane CIC-EC CIE 802, Cayenne General Hospital, Cayenne, French Guiana; 2 EA3593, UFR Médecine - Université des Antilles et de la Guyane, Cayenne, French Guiana; 3 Département des centres de santé, Cayenne General Hospital, Cayenne, French Guiana; 4 Météo-France Cayenne French Guiana; 5 Laboratoire Hospitalo-Universitaire de Parasitologie et Mycologie Médicale, Centre Hospitalier de Cayenne, rue des Flamboyants, BP 6006, F- 97306 Cayenne (French Guiana

## Abstract

**Background:**

The epidemiological profiles of vector-borne diseases, such as malaria, are strongly associated with environmental conditions. An understanding of the effect of the climate on the occurrence of malaria may provide indirect insight into the anopheles mosquito vectors endemic to a particular region. The association between meteorological and hydrographical factors and the occurrence of malaria was studied in a village in French Guiana during an epidemic caused essentially by *Plasmodium vivax*.

**Methods:**

A cohort of confirmed cases of *P. vivax *malaria occurring between 2002 and 2007 was studied to search for an association between the number of new infection episodes occurring each month, mean, maximum and minimum monthly temperatures, cumulative rainfall for the month and the mean monthly height of the river bordering the village, with the aid of time series. Cross-correlation analysis revealed that these meteorological factors had large effects on the number of episodes, over a study period of 12 months.

**Results:**

Climatic factors supporting the continuance of the epidemic were identified in the short-term (low minimum temperatures during the month), medium-term (low maximum temperatures two months before) and long-term (low maximum temperatures nine months before and high lowest level of the river 12 months before). Cross-correlation analysis showed that the effects of these factors were greatest at the beginning of the short rainy season.

**Conclusion:**

The association between the river level and the number of malaria attacks provides clues to better understand the environment of malaria transmission and the ecological characteristics of the vectors in the region.

## Background

Malaria may be seen as the combination of a Plasmodium, an Anopheles mosquito and a human host. Factors modifying the presence, survival or abundance of any one of these actors are likely to favour the maintenance or breaking of the parasite cycle. Environmental conditions are among the factors likely to affect this parasite cycle. The temperature of the water collecting in hollows, the presence of vegetation or predators and altitude may all favour Anopheles mosquitoes or limit their development within a given geographic zone [1,2].

The ecological characteristics of the vectors of malaria in French Guiana have not been completely elucidated [3,4]. In the absence of entomological data, the goal of this study was to investigate the environmental factors conditioning the encounter between humans and parasites, focusing on the incidence of malaria episode in the village of Cacao in French Guiana. Over a period of 14 years, there were no cases of malaria in this village. Then, between 2002 and 2007, an epidemic caused essentially by *Plasmodium vivax *occurred, affecting almost 43% of the inhabitants of the village.

To better understand the determinants of this epidemic, this manuscript presents results studying the association between meteorological and hydrographical factors and the occurrence of malaria among the inhabitants of Cacao.

## Methods

A cohort of patients from the Cacao health centre presenting between January 1^st ^2002 and December 31^st ^2007 with an episode of vivax malaria confirmed by biological tests was studied. The only health facility within a radius of 80 kms was located in the village. A malaria case corresponding to an attack of malaria was defined as an episode of fever (fever recorded or history of fever in the previous 48 hours, associated to the finding of asexual forms of Plasmodium in the blood using Giemsa-stained thick and thin blood smear. in the absence of any other clear aetiology. The parasitological results were validated by a biologist from Cayenne University Hospital Parasitology and Mycology Laboratory (LHUPM). Only patients residing permanently in the village during the study period were included. To ensure data quality, the population that was living temporarily in Cacao (seasonal workers, visitors, tourists...) was not included in the analysis. Therefore, the sample considered in the analysis, remained stable. Patients were included in the cohort from the day the positive sample for *Plasmodium vivax *was obtained. The primary endpoint was the occurrence of a new episode of *P. vivax *malaria (detected by the health centre in Cacao and subsequently biologically confirmed, excluding cases of treatment failure). Uncomplicated malaria cases were treated with chloroquine. None of the patients received 8-aminoquinoline. Relapses were excluded by applying the rule of three months as shown recently in French Guiana for local strains of *P. vivax*. Indeed, in a study carried out in Camopi, a village located in an endemic area, the secondary *P. vivax *attack was considered as a relapse when it occurred during the first three months after the first attack [5].

The association between climatic factors and the monthly incidence of *P. vivax *malaria was investigated by a time-series model of the autoregregressive integrated moving average (ARIMA) type. The series was non-stationary and the variance grew exponentially. First, logarithmic transformation ensured the normality and homogeneity of the variance of the residuals. Then, to make the series stationary, a first-order differentiation was performed [6]. The parameters of the ARIMA and the seasonal parameters were determined with the aid of Akaike's information criterion (AIC), the autocorrelation function (ACF) and the partial autocorrelation function (PACF). The model chosen was ARIMA(1,1,1)(0,1,1)_12_. The ARIMA residuals were not (auto-) correlated and were normally distributed according to the residuals plot and the autocorrelogram of residuals. The Ljung-Box Q test was also applied to confirm that the residual series were white noise.

**Figure 1:**
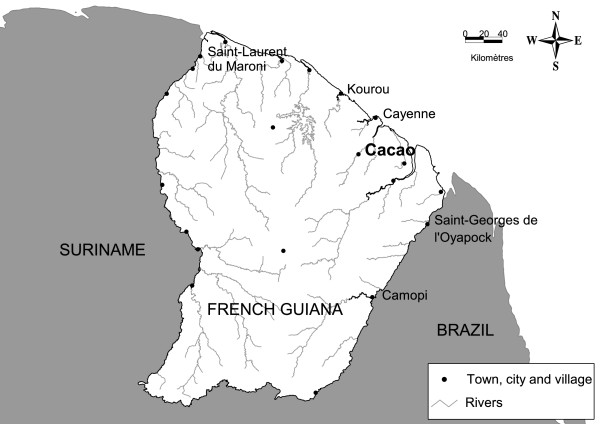
**Localization of Cacao in French Guiana**.

The meteorological factors studied (measured at the Météo France weather station at the village) were selected on the basis of the specific climatic features in the study zone. This zone has a humid tropical environment and is covered by a dense forest with large amounts of aerial biomass. Humidity is about 90% all year round. The village of Cacao is situated about 80 km east of Cayenne, the main city of French Guiana (Figure 1). The mountain tops are low, at an altitude of only 30 to 400 m. The village of Cacao is bordered to the North West by a river, the Comté. The region has four seasons: i) a short rainy season (November to January); ii) a "short summer" (February and March); iii) a long rainy season (mid-March to mid-July), and iv) the dry season (August to November).

The following factors were studied: i) mean minimum temperature for each month; ii) mean maximum temperature for each month; iii) cumulative rainfall for each month; iv) the lowest level of the Comté for each month and v) the highest level of the Comté for each month. These climatic data were measured daily and introduced into the model in the form of the mean monthly value for a period of up to 12 months. These factors were tested in the univariate analysis, with all factors identified as significant at the 10% level being introduced into the multivariate model. A stepwise descending approach was used to obtain the final model. The residuals of the models were visualised graphically (by autocorrelation and non systematic tendency). Spearman's rank correlation analysis was carried out to assess the association between the occurrence of malaria attacks and meteorological factors.

Statistical significance was set at p < 0.05. The data was analyzed using STATA 10.0 (STATA Corporation, College Station, Texas).

## Results

**Figure 2:**
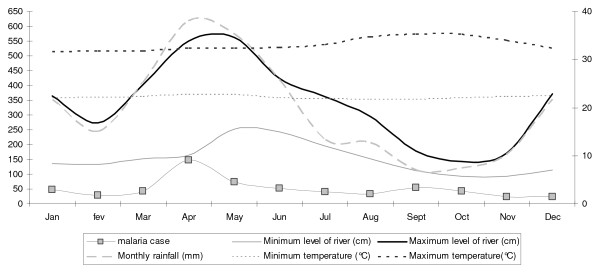
**The incidence of malaria was highest in 2005 and 2006 (figure 2a); the Comté reached its highest level between 2002 and 2007 in May 2002, when it reached a height of 6.4 m, and its lowest level in December 2004, at 75 cm (figure 2b)**.

The village of Cacao is inhabited by 839 people, 359 of whom presented at least one attack of *P. vivax *malaria between 2002 and 2007. The Cacao health centre detected 817 attacks of *P. vivax *malaria and 40 attacks of *Plasmodium falciparum *malaria among the inhabitants of this village during this epidemic. The incidence of attacks due to new infections (616 episodes) followed a seasonal pattern over the 12 months of the year, with a peak in April (Figure 2).

Between 2001 and 2007, the months of March, April, May and June had the highest levels of rainfall during the year (cumulative rainfall levels greater than 400 mm per month) and, in these months, the height of the river exceeded 4 m. Minimum and maximum temperatures remained fairly stable, with a slight increase in maximum temperatures observed between August and November.

**Figure 3:**
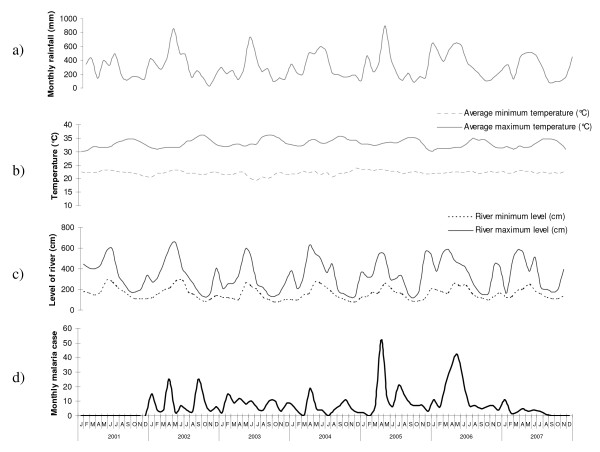
**Between 2001 and 2007, the mean cumulative rainfall per month (a), mean monthly maximum and minimum temperatures (b), the mean level of the river Comté (c) and the number of *P. vivax *malaria attacks per month at Cacao (d)**.

The mean minimum temperature fell below 21°C between June and September 2003, reaching a trough of 19.4°C in July 2003. The mean maximum temperature exceeded 35°C during the following periods: September and October 2002, from August to November in 2003 and September and October in 2004 and 2005, with a peak at 36.3°C during August 2003 (Figure 3b). Precipitation levels followed a strongly seasonal pattern (Figure 3a) during the study period. Rainfall levels were highest in April 2002, May 2003 and April 2005 (cumulative precipitation of 893.2 mm for this month).

The introduction of meterological factors into the univariate ARIMA model (1,1,1)(0,1,1)_12 _identified nine relevant factors: i) mean minimum temperatures at time t and t-12 months (factor 1 and 2); ii) mean maximum temperature at t-1, t-2 and t-9 months (factor 3,4,5); iii) the minimum level of the Comté at t-11 and t-12 months (factor 6 and 7); and iv) the maximum level of the Comté at t-8 and t-12 months (factor 8 and 9).

The multivariate ARIMA model obtained with the inclusion of these nine variables, applying a stepwise descending procedure, is summarized in Table 1. The incidence of a malaria episode at time t was positively correlated with the minimum level of the Comté at t-12 months and inversely correlated with the minimum temperature at time t and with maximum temperature at times t-2 and t-9 months.

**Table 1:** ARIMA regression model of the logarithm of monthly malaria cases (2001-2007) against meteorological factors in Cacao, French Guiana

	**MODEL (AIC = 145.23)**		
	
	**Coefficient**	**SEM**	**p-value**
	
Minimum temperature	-0.272	0.15	0.007
Maximum temperature (lag 2)	-0.307	0.13	0.002
Maximum temperature (lag 9)	-0.312	0.098	0.001
Minimum level of river (lag 12)	0.0091	0.0032	0.005
AR	-0.084	0.176	0.635
MA	-0.719	0.098	0.000
Seasonal MA	-0.505	0.13	0.000
Constant	0.037	0.015	0.012

AR: autoregressive term MA: moving average term

**Figure 4:**
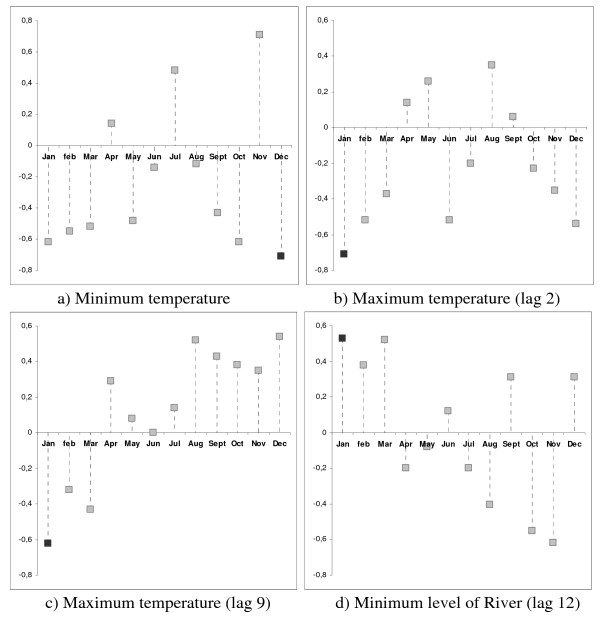
**Cross-correlations between meteorological factors and malaria cases in Cacao from 2001 to 2007**.

The relationship between the incidence of malaria and the minimum temperature (Figure 4a) was strongest in December (rho = -0.7143; p = 0.1108). Correlations between the number of attacks and maximum temperatures two months before (Figure 4b) and nine months before (Figure 4c) were strongest in January (rho = -0.71; p = 0.117 and rho = - 0.62; p = 0.1911, respectively). The same was true for the minimum level of the Comté river (Figure 4d; rho = 0.53; p = 0.27)

## Discussion

This study was carried out on a small cohort, limiting the power of statistical analyses, including cross-correlations in particular. However, it should be noted that the medical data obtained are exhaustive for the village, homogeneous (malaria caused by *P. vivax *only) and high quality (identical diagnostic and therapeutic management over a period of five years).

Very little entomological knowledge is currently available for the vector of malaria at Cacao [3]. Vector control programmes are, therefore, based on data from the other countries of South America, for *Anopheles darlingi *[7-11]. The lack of local specificity of the knowledge regarding the vector results in intervention programmes needing to be massive, with poor cost/efficacy ratios.

This study could indirectly help to improve this ratio, by providing clues to better understand the transmission of the parasite between vector and host in this region. Indeed, the results of this study showed certain climatic factors could participate in the maintenance of the epidemic in the short-, medium- or long-term.

First, the positive association between the river level and the incidence of malaria could be explained by the location of potential larval habitats. The banks of the Comté river are encumbered with voluminous tree roots and are, therefore, not straight. During periods of high water, these features favour the creation of pockets of water with weak currents, bordered by vegetation and ideal for the development of anopheles mosquito larvae [11-13]. The persistence of a minimum level of water in the river ensures the maintenance of these larval habitats and adequate development of the surrounding vegetation. Without entomological evidence, this hypothesis could not be confirmed. However, others authors in America have also proposed this. In the north of Peru, a number of climatic factors (temperature and rainfall figures) have been studied, but only the level of the river Nanay was found to be significantly associated with the incidence of malaria due to *P. vivax *[14]. Similarly, a study in Suriname identified associations between mosquito density and the level of the river [15]. In Brazil, flooding of the fields bordering the river Branco has also been suggested as a reason for increases in the number of *An. darlingi *bites per person during the rainy season, close to the river [16]. In Venezuela, the abundance of *An. darlingi *was positively correlated with the maximum level of the Orinoco river because of the creation of lagoons during the river overflow [17].

The time lag between the occurrence of malaria and the associated meteorological factors, such as maximum temperature nine months previously and the level of the Comté 12 months previously, would suggest that meteorological conditions in a given year may affect malaria in the following year. With persistent larval habitats in all seasons due to sufficient river levels and low temperatures, the transmission of malaria is maintained into the next year. These environmental factors could create the appropriate conditions to preserve mosquito larvae with sufficient humidity in the dry season. Another hypothesis would be the need for a number of parasitic amplification cycles to make malaria transmission patent. The long-term impact of climate on malaria has already been reported in the literature. Indeed, a seven-month delayed effect of fog was associated with malaria incidence in a Chinese study. The authors explained this delay with the same biological hypothesis [6]. Similarly, significant increases of malaria mortality and morbidity have been showed in years following recognized El Niño events [18].

The intertropical convergence zone (ITCZ) or meteorological equator determines the seasons in French Guiana. It descends the northern hemisphere, crossing French Guiana in mid-December and bringing with it cumulonimbus clouds, which generate rainfall. Cross-correlation analysis showed that the time at which temperatures and the level of the Comté river seemed to influence the occurrence of cases coincided with the period during which the ITCZ passes through French Guiana for the first time. The resulting increase in precipitation, with high humidity, favours an increase in the number of mosquito breeding sites and the development of the aquatic stages of the mosquito [19].

Finally, an increase in temperature (both minimum and maximum) has often been identified as a factor accelerating the gonotrophic cycle of anopheles mosquitoes, decreasing the interval between egg-laying episodes and increasing vector abundance [20-23]. The development cycle of the parasite is also accelerated by high temperatures. However, if the temperature is too high, it may also jeopardise the survival of adult mosquitoes and dry up breeding sites more rapidly, particularly during the dry season. Indeed, in this analysis, the number of malaria cases in 2003 and 2004 fell sharply when temperatures were particularly high.

## Conclusion

A better understanding of the ecology of the anopheles mosquitoes endemic to the environment should make it possible to concentrate human and material resources more effectively in vector control programmes. In the absence of entomological and behavioural data, analyses of the incidence of malaria over time has provided information about the transmission of *P. vivax *in the region, and this information may be useful for the prevention and control of future epidemics.

## Competing interests

The authors declare that they have no competing interests.

## Authors' contributions

CB participated in the research design, performed data analysis and interpretation, and prepared the manuscript. MH participated in the statistical analysis. RHS, FG, CG and MJ participated in interpretation of data and manuscript revision as well. SG participated in research method. MN provided guidance on data analyses and was involved in the interpretation of data and manuscript revision. BC was responsible for data collection, initiated the study, and was involved in the interpretation of data and manuscript revision. All authors read and approved the final manuscript.
